# Post-Adoption Help-Seeking in Portugal: A Comprehensive Study on Parental Difficulties and Help-Seeking Behaviors and Perceptions

**DOI:** 10.3390/ijerph191811283

**Published:** 2022-09-08

**Authors:** Stephanie Alves, Ana Luz Chorão, Bárbara Caetano, Margarida Rangel Henriques, Isabel Pastor, Raquel Pires

**Affiliations:** 1 ProChild CoLAB against Child Poverty and Social Exclusion, Campus de Azurém, Edifício 1, 4804-533 Guimarães, Portugal; 2 Faculty of Psychology and Education Sciences, University of Coimbra, Rua do Colégio Novo, 3000-115 Coimbra, Portugal; 3 Cognitive Behavioral Clinical Psychology Unit (UpC3), Faculty of Psychology and Education Sciences, University of Coimbra, Rua do Colégio Novo, 3000-115 Coimbra, Portugal; 4 Center of Psychology, Faculty of Psychology and Sciences of Education of the University of Porto, University of Porto, Rua Alfredo Allen, 4200-135 Oporto, Portugal; 5 Santa Casa da Misericórdia de Lisboa, Largo Trindade Coelho, 1200-470 Lisbon, Portugal; 6 Center for Research in Neuropsychology and Cognitive Behavioral Intervention, Faculty of Psychology and Education Sciences, University of Coimbra, Rua do Colégio Novo, 3000-115 Coimbra, Portugal

**Keywords:** post-adoption, parents, difficulties, help-seeking behaviors, perceived barriers to seeking help, parenting interventions, acceptability

## Abstract

Effective psychological parenting interventions delivered to adoptive parents may prevent serious adjustment difficulties and promote a healthy functioning; however, less is known about adoptive parents’ specific parental difficulties and help-seeking behaviors and perceptions, the understanding of which is deemed necessary to design well-informed interventions. This study aimed to describe parental difficulties, help-seeking behaviors, satisfaction with, and perceived barriers to seek, professional help, and acceptability of psychological parenting interventions among Portuguese adoptive parents. Comparisons with biological parents (Study 1) and between adoptive parents that requested adoption-specialized and non-specialized support (i.e., adoption-specialized vs. non-specialized help-seekers) (Study 2) were explored. A cross-sectional online survey was conducted. Participants were 471 adoptive and 552 biological parents of children aged under 18 years who were recruited through schools, adoption agencies, and social networks. They completed measures assessing parental difficulties, help-seeking behaviors, satisfaction with, and perceived barriers to seek, professional help, and acceptability of psychological parenting interventions. Comparison tests between the study groups, Pearson’s correlations, analyses of covariance (ANCOVA) and multivariate logistic regressions (MLR) were performed. Parents’ well-being and normative parenting challenges were the most frequent difficulties in both groups, but only associated with adoptive parents’ help-seeking. Although difficulties related to a child’s problems/parent–child relationship were more frequent among adoptive parents, adoption-related difficulties were rarely reported. Adoptive parents sought professional help more frequently, regardless of parental difficulties. Knowledge-related barriers to seeking help were the most frequent among adoptive parents. Adoption non-specialized help was less satisfactory. Acceptability of psychological parenting interventions was high, but dependent on parental difficulties. Implications for post-adoption services’ development are discussed.

## 1. Introduction

Adoptive and biological parents face similar challenges during the transition to parenthood [1]. However, adoptive parents need to manage additional adoption-related issues (e.g., experiences with infertility, increased likelihood of parenting children with preexisting behavioral/emotional difficulties, stigma attached to adoption) [1,2,3], which may make them more vulnerable to parental difficulties [4,5].

There is a grown recognition that some adoptive families will require professional help at some point during the family life cycle [6] and that its provision may promote a successful adjustment and permanency of adoptive families [7,8,9]. However, adoptive parents have reduced contact with adoption services over time [8], making access to post-adoption support dependent on parents’ help-seeking [10,11,12], and little is known about the specific features of help-seeking among this population [13]. Adoptive parents’ reasons for seeking or not seeking professional help [14], their satisfaction with [15] and acceptability of specific types of interventions [16] are some of the understudied topics, which can greatly differ from biological parents [4]. Comparison studies between adoptive and biological families have been highly recommended [3,16], as they would allow a deeper understanding of the influence of the unique post-adoption features on parental difficulties and help-seeking above and beyond what is expected for normative/biological parenthood.

In Portugal, this knowledge is even more urgent. Since 2015, a legal framework for the provision of post-adoption support was set out [17], but no structured governmental post-adoption services are yet available. Adoption-specialized support is only available through valuable, but isolated, social, academic or private sector initiatives, in specific regions of the country. Most adoptive parents relied on generalist services designed to attend biological parents’ needs, rather than adoption-specialized professional help, which may compromise its effectiveness. However, as in other countries, to ensure that Portuguese adoptive families may access the professional specialized support they need, several research questions should be first addressed.

### 1.1. Specific Types of Parental Difficulties

Few studies have compared parental difficulties among adoptive and biological families, and most of them considered global dimensions of parental difficulties and reported contrasting results. For instance, parenting stress has been the main dimension studied across studies exploring differences between adoptive and biological parents; several studies found that adoptive parents reported higher [18,19] or lower [20,21] levels of stress when compared to biological ones. Other studies reported an absence of significant differences [22]. This inconsistency has been considered the result of methodological discrepancies across studies but may also be explained by the lower sensitivity of global measures to capture the specificity of adoptive parental difficulties [4].

Most studies addressing specific parental difficulties focused on child-related stress, showing that adoptive parents perceived more difficulties related to their children’s behaviors and the parent–child relationship than biological parents [18,19,23]. These results are supported by adoption studies that highlighted that adoptive parents frequently report parent–child attachment difficulties [13], concerns regarding their child’s development [24,25], deviant behaviors [13], and emotional, behavioral or medical problems [24,26]. Adoptive parents also reported school-related challenges, namely with school staff or setting, and special education needs [13,24]. Moreover, besides reporting adoption-specific concerns (e.g., talking with children about adoption; supporting children’s curiosity about their origins) [24,27], adoptive parents also express increased normative parenting concerns (e.g., establishment of routines and limits) [27,28]. Less comparative research has paid attention to other areas of parental functioning such as mental health and marital relationship quality [3]. Overall, evidence highlights an advantage for adoptive families, who tend to report fewer mental health difficulties and greater well-being [3,21,29], as well as greater marital relationship quality and family cohesion [21,29], compared to biological ones.

Although all these domains of parental difficulties are known to be related to children’s adjustment and development [3], few comparative studies have analyzed them comprehensively, together in the same sample. Indeed, current research has examined parental difficulties in isolation, which turn difficult to characterize and explore the relative influence of each difficulty in combination. Along to possibly blurring effects, this limits our understanding of which specific domains of parental difficulties represent a greater and/or specific issue of adoptive parenthood.

### 1.2. Help-Seeking: Associated Parental Difficulties and Perceived Barriers

Previous research highlights a mixed picture regarding post-adoption support services [25]. They usually include educational, informational (e.g., seminars, support groups), clinical (e.g., crisis intervention services, counseling) and material (e.g., adoption subsidies, respite care) services [6]. Past research has shown that several families sought post-adoption services over time [13,24] and that, compared to biological parents, they seem to seek professional help more frequently [23,30]. However, although previous literature suggests that adoptive parents seek professional help especially to address their children’s emotional/mental health needs [7,12,25,26], the specific parental difficulties associated with help-seeking remains understudied.

On the other hand, a significant proportion of adoptive families do not seek professional help [10,26,31], even when they say that need it [7], or request it only at a crisis point [12]. This evidence suggests that there are barriers that may prevent adoptive families from seeking professional help to deal with parental difficulties; those barriers can be grouped into knowledge, attitudinal and practical/structural barriers [32,33]. Regarding knowledge barriers, adoptive parents may have difficulties in recognizing their parental difficulties as a problem requiring professional intervention [14]. They may also not identify the need of additional and differential help for their families compared to the one provided to biological ones [34,35]. This poor recognition of support needs is likely to be exacerbated by lack of professional agreement in defining/recognizing a problem that is severe enough to require clinical intervention [36]. Besides, adoptive parents also report having limited knowledge about which post-adoption services are available and where and how they can be accessed [10,11,35,37]; they may also be not aware of their eligibility to receive such services [10,14].

Several attitudinal barriers in obtaining post-adoption services were also identified. Concerns of being perceived as failures as a parent or having their children removed [12,35,36], feelings of stigma [11], and concerns about their adopted children being negatively labelled [35] are among them. Lack of adoption-competent professionals [37,38,39] and perceiving past services as not helpful [11] have also been reported as barriers to seeking post-adoption support.

Finally, practical/structural barriers include geographic distance to services, unavailability or inadequacy of support services, professionals’ turnover, uneven distribution of services, limited access to health care, and time and financial constraints [10,14,35,39]. Despite most of the barriers identified in adoption literature share similarities to what was found with biological parents (e.g., for perinatal depression) [32,33,40], there has been no research comparing adoptive and biological parents on this topic, which hinder a comprehensive picture of the relative importance of the different barriers to help-seeking during the post-adoption period and that should be consequently addressed.

### 1.3. Parents’ Satisfaction and Acceptability of Future Interventions

When exploring adoptive parents’ satisfaction with the services accessed, the results showed mixed findings. A number of studies showed high adoptive parents’ satisfaction with post-adoption services [7,10,31]; others highlighted that some adoptive parents perceived post-adoption services as unhelpful [8,39], often report unmet needs of support [9,25,26], and had concerns with the lack of competent adoption professionals and services [26,39]. For instance, adoptive parents discuss the extent to which many professionals had difficulties in understanding the unique experiences, needs and challenges of adoptive families [15,26] and, therefore, in offering helpful and sensitive advice [38]. In line with this, it is plausible to assume that adoptive parents are less likely to be satisfied with the support they received when compared with biological ones [4], namely if this was a non-specialized support.

Moreover, a common need that remains overlooked by many post-adoption services is the well-being of adoptive parents. Mental health problems are commonly experienced by adoptive parents [3,13] and had well-established consequences on parent–child relationship and children’s outcomes [4]. However, adoptive parenting intervention programs focus primarily on improving children’s well-being rather than parents’ outcomes [5,16]. Interventions that target adoptive parents’ well-being and other well-established mechanisms of change underlying the outcomes of available parenting interventions (e.g., parent–child relationship) [4] may be one promising avenue to address adoptive families’ needs. Based on prior conceptualizations in the adoption [16] and parenting [40,41] literature, we used the term “psychological parenting interventions” in this study to encompass a range of interventions based on psychological theories, directed at parents only or including the child, and targeting various features related to the parental role (e.g., parental mental health, parent–child relationship). They can include a variety of delivery formats and features and have shown to improve parents and child outcomes [16,41].

Previous research has highlighted the greater acceptability of psychological parenting interventions among adoptive rather than biological parents [4], though focused on children’s rather than parents’ own needs. Besides, most studies have assessed acceptability after rather than before intervention delivery [16], focusing more on the behavioral component of program engagement (e.g., attendance rates) as opposite to the attitudinal one [42], which can encompass perceived intervention benefits/utility [42] and intention to participate [43]; this lack of knowledge limited our understanding of more nuanced factors likely to influence parental initial intention to engage in parenting interventions (e.g., parents’ psychological distress, difficulties in the parent–child relationship [43]), which could offer insights for intervention’s development and dissemination. Accordingly, further research is needed to carefully assess the acceptability of such interventions to adoptive parents [4,16] as well as its potential correlates (e.g., specific parental difficulties). In line with similar studies [40], acceptability was defined in the present study as comprising the parents’ perceived utility and availability to participate in future psychological parenting interventions targeting their own well-being or the parent–child relationship.

### 1.4. The Present Study

In the present study, we intended to examine specific types of parental difficulties among Portuguese adoptive parents and their associated help-seeking behaviors and perceptions (i.e., perceived barriers, satisfaction and acceptability), by identifying the extent to which they: (1) differ from those presented by biological parents (Study 1), and (2) vary according to the adoption-specialized or non-specialized nature of the professional help asked by adoptive parents (Study 2).

In study 1, we first hypothesized that, when compared to biological parents, adoptive parents would report more difficulties concerning (1) their children’s physical health problems, (2) their children’s developmental/emotional/behavioral problems, (3) the parent–child relationship and (4) normative parenting challenges, and less difficulties regarding (5) their own well-being, (6) the couple relationship and (7) co-parenting. We also expected that adoptive parents would experience (8) adoption-related difficulties. Regarding help-seeking behaviors, we hypothesized that (9) adoptive parents sought professional help more frequently than biological ones and that the parental difficulties that would be more associated with help-seeking may follow the trend of the most frequent parental difficulties in each group; that is, parental difficulties related to (10) children’s physical health problems, (11) children’s developmental/emotional/behavioral problems, (12) the parent–child relationship and (13) normative parenting challenges would be associated with help-seeking behaviors among adoptive parents, while difficulties related to (14) parents’ own well-being, (15) the couple relationship and (16) co-parenting would be associated with help-seeking behaviors among biological parents. Second, we hypothesized that adoptive parents would report (17) higher knowledge and attitudinal barriers, (18) less structural/practical barriers and (19) less satisfaction with the professional help received than biological ones. Given the lack of literature on these topics, we did not elaborate hypotheses regarding the association between perceived barriers nor the satisfaction with the professional help received and specific types of parental difficulties. Finally, we hypothesized that adoptive parents would report (20) higher acceptability (i.e., higher perceived utility and availability to participate) of future psychological parenting interventions aimed to promote parental well-being and the quality of parent–child relationship compared to biological ones and that (21) greater parental difficulties regarding parents’ own well-being and the parent–child relationship would be associated with higher acceptability of psychological parenting interventions among adoptive parents.

In Study 2, we hypothesized that, compared with adoptive parents who sought non-specialized professional help, parents who asked for adoption-specialized help would report (1) higher adoption and (2) child-related difficulties (i.e., children’s developmental/emotional/behavioral problems, children’s physical health problems, children’s deviant behavior, and children’s sexualized behavior), as well as (3) higher satisfaction with the professional help received. Given the lack of literature on these topics, we did not formulate hypotheses regarding the acceptability of future psychological parenting interventions according to the type of help-seeking nor regarding the associations between parents’ satisfaction with the professional help received, their acceptability of future psychological parenting interventions and specific types of parental difficulties.

## 2. Materials and Methods

### 2.1. Procedures

This cross-sectional study is part of a larger project hosted in, and approved by the Ethics Committee of, the Faculty of Psychology and Educational Sciences of the University of Coimbra, Portugal; it was carried out with the collaboration of scientific and community institutions acting in the fields of adoption and parenting, and all the Portuguese adoption agencies. Data were collected between September 2020 and April 2021 through an online self-report survey. A secure online survey tool provided by the host institution (LimeSurvey) was used.

Participants were recruited through adoption agencies, schools and social networks. Potential participants were invited to participate in the study through an email sent by the schools and adoption agencies, which contained summary information about the objectives of the study and the researchers’ contacts, as well as the link to the online survey. Specifically, regarding recruitment via adoption agencies, the main researcher met with the Portuguese governmental adoption agencies (i.e., the Institute of Social Security, I.P., the Institute of Social Security of Madeira, IP-RAM, the Institute of Social Security of the Azores, the Santa Casa da Misericórdia de Lisboa) to explain the study and the procedures of contact with each potential participant; therefore, the agencies disseminated the research project among adoption professionals by email and required their collaboration in participants’ recruitment by sending the template e-mail referred above to each adoptive family with whom they have contacted before. On social networks, the link to the survey was periodically posted on the research project’s Facebook page and freely disseminated by other pages interested in its divulgation.

The eligibility criteria were: (1) being 18 years or older and (2) had at least an adoptive or a biological child under 18 years old. The participation in this study was voluntary. Anonymity and confidentiality were ensured. Informed consent was electronically obtained from all participants by clicking on the option “yes, I authorize” after reading the full information about the research project, the inclusion criteria, the investigator’s duties, the participant’s rights and the data protection policy used for data storage. Parents who had more than one child under 18 years were then instructed to report on the child with whom they felt more difficulties.

Of the 1412 responses to the survey (694 adoptive and 718 biological parents), 389 parents (223 adoptive and 166 biological parents) were excluded due to one or more of the following reasons: (1) if children were more than 18 years old; (2) if missing information in one or more main study variables; (3) if parents with adoptive and biological children reported to the biological child as whom they experienced more difficulties; and (4) if both members of the couple completed the questionnaires, only one of them was randomly included in the present study (in order to ensure the independence of the observations).

### 2.2. Sample

The sample comprised 1023 Portuguese parents aged between 21 and 66 years old (independent observations). In Study 1, this sample was divided into two groups, according to family type: 471 adoptive and 552 biological parents. In study 2, we used a subsample of adoptive parents who asked for professional help (i.e., help-seekers adoptive parents); they were divided into two subgroups, according to the type of professional help asked for: 82 adoptive parents that requested adoption-specialized support (i.e., adoption-specialized help-seekers) and 118 adoptive parents that requested non-specialized support (i.e., non-specialized help-seekers).

As depicted in Table 1, adoptive parents were, on average, 47 years old. Most of them had a university/postgraduate degree, were married, employed, and never had physical or mental health problems. Compared with biological parents, they were older, reported being employed with more frequency, and there were more adoptive fathers in the sample; adoptive parents were less likely to have a university/postgraduate degree and a history of mental health problems, and they perceived a more positive impact of COVID-19 in their lives. Adopted children had a mean age of 10 years old, being older than children of biological parents.

On average, adoptive parents had adopted a single child, in most cases as a couple (see Table 1). All the adoptive parents who had biological children identified the adoptive child as the child with whom they felt greater difficulties or concerns, 100%, *n* = 84. There were no international adoptions in our sample. Adoptive parents have adopted their children, on average, 6 years before this study and the children’s mean age at adoptive placement (i.e., the moment that the child starts to live with his/her new adoptive family) was 4 years old. Detailed information for the subsample of help-seeker adoptive parents (Study 2) is presented in Table 2. Children from adoption-specialized help-seekers were, on average, older than children from non-specialized help-seekers.

### 2.3. Measures

An online survey was developed specifically for this study, based on an exhaustive literature review in the field of adoption, parenting, help-seeking and parenting interventions, as well as on instruments already used in similar surveys [32,40]. The survey had two equivalent versions: one for adoptive parents and another for biological ones. A first version of the survey was revised by key stakeholders (i.e., academics and professionals) in the adoption and parenthood fields and subsequently pilot tested using a small sample of biological and adoptive parents and adjusted to ensure clarity and comprehension.

#### 2.3.1. Sociodemographic, Health and Adoption-Related Data

Regarding sociodemographic data, parents were asked about their age, sex, education, professional status, marital status, number of household members, family type and number of biological and/or adopted children. Regarding health-related data, parents were asked about their history of physical and mental health problems. As sample collection occurred during the COVID-19 pandemic, participants were also asked about COVID-19 infection and if they were part of the risk population for COVID-19. COVID-19 perceived impact was also evaluated, by asking participants to answer the question “Please, rate the impact that COVID-19 pandemic had/has in each one of the following areas of your life”, using a 5-point scale (from 1 = *Very negative impact* to 5 = *Very positive impact*). The items covered (1) physical health, (2) psychological health, (3) financial/employment stability, (4) relationship with friends, (5) relationship with extended family, (6) relationship with the partner, (7) relationship with the child(ren) and (8) parental role. A total score was obtained through the average of all items, with higher scores indicating a more positive perceived impact and lower scores reflecting a more negative perceived impact. Cronbach’s alpha was 0.73 and 0.74 for adoptive and biological parents, respectively. Parents also reported data related to their children (i.e., age and sex) and the adoption process, if applicable (i.e., number of children adopted at the same time, application type, children’ age at adoptive placement and years since the adoptive placement has occurred).

#### 2.3.2. Parental Difficulties

A set of items was formulated to operationalize specific parental difficulties reported in the literature as common among adoptive parents [13,24]. This scale was developed in order to be suitable for application to both adoptive and biological parents. Key stakeholders in the adoption field were asked to indicate the extent to which they consider that the items accurately captured the difficulties that adoptive parents may experience. The final version of this questionnaire included parental difficulties related to: (1) parents’ well-being (e.g., mental health, stress, capacity and/or energy to manage daily demands); (2) parent–child relationship (e.g., emotional bonds, communication, conflict resolution); (3) normative parenting challenges (e.g., understanding of the child’s development and needs, establishment of educational strategies, bounds and routines); (4) couple relationship; (5) co-parenting; (6) children’s developmental/emotional/behavioral problems (e.g., developmental delays, anxiety, depression, behavior problems, poor academic achievement, siblings’ relationship, socialization with other children); (7) children’s deviant behavior (e.g., lies, theft, serious aggression); (8) children’s sexualized behavior; (9) children’s physical health problems (e.g., disabilities or chronic health conditions); (10) communication with external contexts to the family (e.g., school); and (11) adoption-related difficulties (e.g., communication about adoption, separation from the biological family, search for origins, cultural differences, the child’s life story and its consequences). With the exception for the last item, all items were answered by adoptive and biological parents, on a 7-point scale (from 0 = *Never* to 6 = *Always*). Cronbach’s alpha was 0.89 and 0.78 for adoptive and biological parents, respectively. Parents were also asked to identify how long after adoptive placement (adoptive parents)/birth (biological parents) they experienced the most severe parental difficulties (response categories ranged between a minimum of “during the first year after adoptive placement”, and a maximum of “more than 10 years after adoptive placement”).

#### 2.3.3. Help-Seeking Behaviors and Perceptions

Help-seeking behaviors were assessed through the questions “Have you sought professional help (e.g., psychological, psychiatric, another medical support or support from adoption professionals) for dealing with parental difficulties?” and “Was this professional help specialized in adoption issues?” (*yes* versus *no*).

To assess perceived barriers to seeking professional help, a set of items were developed based on the Barriers Scale [33] and general literature on the transition to parenthood [32,40], taking into consideration adoption-specific help-seeking features [11,14]. Parents were questioned about the extent to which each item (barrier) prevented them from seeking professional help for their parental difficulties. The 14 items covered knowledge (e.g., “I don’t know where to look”), attitudinal (e.g., “I am afraid they think I am not a good parent”) and structural/practical (“I am not able to afford professional help”) barriers. Participants’ responses were coded as 0 (*Does not apply to me*) or 1 (*Applies to me*).

Satisfaction with the professional help received was inferred from parents’ responses to the question “To what extent do you think the professional help received was helpful?”. This question was answered on a 6-point scale (from 0 = *Not useful* to 5 = *Extremely useful*).

The acceptability of future psychological parenting interventions was inferred from parents’ responses on the questions “Do you think that psychological interventions with the aim to help parents in dealing with difficulties in the relationship with their child or their own well-being as a parent would be useful?” (i.e., perceived utility) and “Would you be available to participate in a psychological intervention program of this scope?” (i.e., availability; *yes* versus *no*).

### 2.4. Data Analysis

Analyses were conducted using the Statistical Package for the Social Sciences, v25 (IBM Corp. in Armonk, NY, USA), considering a significance level of *p* < 0.05. For sample characterization, descriptive statistics, and comparison tests (*t* and chi-square) between the study groups (i.e., study 1: adoptive vs. biological parents; study 2: adoption-specialized vs. non-specialized help-seekers) were performed.

To identify the extent to which adoptive parents’ difficulties and their help-seeking behaviors and perceptions differ from those presented by biological parents (Study 1) and vary according to the adoption-specialized or non-specialized nature of the professional help asked for (Study 2), we performed analyses of covariance (ANCOVA) and multivariate logistic regressions (MLR). Estimated marginal means, standard errors (ANCOVA) and frequencies (MLR) were reported, as well as the significance of group effects on the dependent variables, while controlling for significant differences between the groups. Associations between different types of parental difficulties and parents’ help-seeking behaviors and perceptions were explored in each study group using Pearson’s correlations.

## 3. Results

### 3.1. Study I: Adoptive vs. Biological Parents

#### 3.1.1. Parental Difficulties

Parental difficulties faced by adoptive parents were related, from the most frequent to the less frequent one, to parents’ well-being, normative parenting challenges, parent–child relationship, children’s developmental/emotional/behavioral problems, couple relationship, co-parenting, children’s deviant behavior, communication with external contexts to the family, adoption-related issues, children’s physical health problems and children’s sexualized behavior (see Table 3). Regarding adoption-related difficulties, 244 parents (52.8%) never faced it, 165 (35.1) said they rarely experienced it, 57 (12.1%) had faced it sometimes and only 5 (1%) reported experiencing it almost always/always. For 59.8%, *n* = 150, of the adoptive parents, the most severe parental difficulties emerged during the first year after adoptive placement.

Compared with biological parents, adoptive ones reported less difficulties related to the couple relationship and co-parenting. However, they experienced difficulties related to the parent–child relationship, child’s developmental/emotional/behavioral problems, children’s deviant behavior, children’s sexualized behavior, children’s physical health problems and communication with external contexts to the family more frequently than biological parents. The groups did not differ in difficulties related to parents’ well-being and normative parental challenges, the two most frequent types of parental difficulties in both groups (see Table 3). There was a group effect on the onset of the most severe difficulties, *B* = 1.67, *SE* = 0.25, *p* < 0.001, with more adoptive parents reporting it during the first year after adoptive placement, *n* = 150, 59.8%, than biological parents, *n* = 147, 42.4%.

#### 3.1.2. Help-Seeking: Associated Difficulties and Barriers

About 42%, *n* = 200, of adoptive parents asked for professional help to deal with parental difficulties. This proportion was significantly higher, *B* = 1.31, *SE* = 0.25, *p* < 0.001, than that found among biological parents, *n* = 149, 27%, even after controlling for parental difficulties, *B* = 0.82, *SE* = 0.32, *p* = 0.01. Parental difficulties significantly associated with adoptive parents’ help-seeking were related to parents’ well-being, *r* = 0.15, *p* = 0.02; normative parental challenges, *r* = 0.15, *p* = 0.02; children’s developmental/emotional/behavioral problems, *r* = 0.29, *p* < 0.001; children’s deviant behavior, *r* = 0.22, *p* < 0.001; and communication with external contexts to the family, *r* = 0.13, *p* = 0.04. Among biological parents, only parental difficulties related to children’s developmental/emotional/behavioral problems, *r* = 0.27, *p* < 0.001; deviant behavior, *r* = 0.12, *p* = 0.02; physical health problems, *r* = 0.12, *p* = 0.03; and communication with external contexts to the family, *r* = 0.15, *p* = 0.01, were associated with help-seeking.

The three most frequent barriers for adoptive parents seeking professional help were the normalization of their difficulties, doubts about the severity of their difficulties and not knowing who could help them deal with these difficulties (see Table 4).

#### 3.1.3. Help-Seeking: Satisfaction and Acceptability

On average, both adoptive, *M* = 4.07, *SD* = 0.08, and biological parents, *M* = 3.90, *SD* = 0.10, considered the professional help received as very useful; no significant differences were found between them. Among adoptive parents, satisfaction was negatively associated with parental difficulties related to children’s problems, *r* = −0.14, *p* = 0.04. Among biological parents, the only type of difficulty associated with satisfaction was those related to co-parenting, *r* = −0.20, *p* = 0.03.

Adoptive parents also reported a high perceived utility of, 95.3%, *n* = 449, and availability to participate in, 62.0%, *n* = 292, future psychological parenting interventions aimed to promote their own well-being and the quality of the parent–child relationship. When compared with biological parents, there were no significant differences regarding the perceived utility of these psychological parenting interventions; despite being an adoptive parent was associated with a greater availability to participate, *B* = 0.41, *SE* = 0.17, *p* = 0.02, this effect turned non-significant after controlling for parental difficulties.

Among adoptive parents, higher perceived utility of future psychological parenting interventions was associated with greater difficulties related to their own well-being, *r* = 0.01, *p* = 0.03, the parent–child relationship, *r* = 0.12, *p* = 0.01, normative parenting challenges, *r* = 0.12, *p* = 0.01, children’s deviant behaviors, *r* = 0.11, *p* = 0.02, and communication with external contexts, *r* = 0.15, *p* < 0.001, while among biological parents there was only one significant correlation between the perceived utility of future psychological parenting interventions and parental difficulties related to parents’ well-being, *r* = 0.10, *p* = 0.02. Among adoptive parents, all the types of parental difficulties were positively associated with their availability to participate in such interventions (ranging from *r* = 0.34, *p* < 0.001 for parents’ well-being to *r* = 0.12, *p* = 0.01 for children’s sexualized behavior). Among biological parents, except for difficulties related to the couple relationship, children’s deviant behaviors and physic health problems, all correlations were also significant (ranging from *r* = 0.17, *p* < 0.001 for child’s developmental/emotional/behavioral problems, to *r* = 0.09, *p* = 0.04 for children’s deviant behaviors).

### 3.2. Study 2: Adoption-Specialized vs. Non-Specialized Help-Seekers

#### 3.2.1. Parental Difficulties

Parental difficulties faced by adoption-specialized help-seekers were related, from the most frequent to the less frequent one, to parents’ well-being, children’s developmental/emotional/behavioral problems, normative parenting challenges, parent–child relationship, children’s deviant behavior, couple relationship, co-parenting, communication with external contexts to the family, adoption-related difficulties and children’s physical health problems, and children’s sexualized behaviors (see Table 5). Parental difficulties related to parents’ well-being and adoption-related difficulties were more frequent among adoption-specialized help-seekers than among non-specialized help-seekers. No other significant differences were found. Overall, for 69.5%, *n* = 57, of the adoption-specialized help-seekers, the most severe parental difficulties emerged during the first year after adoptive placement. There were no significant differences between them and non-specializes help-seekers, *n* = 64, 54.2%.

#### 3.2.2. Satisfaction and Acceptability

Adoption-specialized help-seekers considered the professional support received as more useful, *M* = 4.24, *SD* = 0.11, than non-specialized help-seekers, *M* = 3.90, *SD* = 0.09, *F* = 5.98, *p* = 0.02. While among non-specialized help-seekers there were no significant associations between their satisfaction and specific types of parental difficulties, among adoption-specialized help-seekers there was a negative association between parents’ satisfaction and difficulties related to the quality of parent–child relationship, *r* = −0.26, *p* = 0.02.

Adoption-specialized help-seekers reported a high perceived utility of, 98.8%, *n* = 81, and availability to participate in, 84.1%, *n* = 69, future psychological parenting interventions aimed to promote their own well-being and the quality of the parent–child relationship, as well as non-specialized help-seekers. There were no significant group effects on the perceived utility, nor the availability to participate. For both groups, no significant associations were found between participants’ perceived utility of future parenting psychological interventions and specific types of parental difficulties. While among non-specialized help-seekers there was only a significant association between their availability and difficulties related to their own well-being, *r* = 0.19, *p* = 0.04, among adoption-specialized help-seekers there was a positive association between their availability to participate and difficulties related to their own well-being, *r* = 0.23, *p* = 0.04, and their children’s problems, *r* = 0.28, *p* = 0.01.

## 4. Discussion

The aims of this study were to examine specific types of parental difficulties and help-seeking behaviors and perceptions among Portuguese adoptive parents, and to compare them with biological parents (Study 1) and by type of help-seeking (Study 2). Several of the main innovative findings on these topics will be discussed.

### 4.1. Parental Difficulties

The hypotheses that adoptive parents would present less frequent difficulties related to parents’ well-being and higher difficulties on normative parenting challenges than biological ones were not supported. Rather, both groups of parents reported high and similar levels of difficulties in these domains. These findings are congruent with previous studies suggesting that emotional problems may be common among adoptive parents, namely due to the mismatch between parents’ expectations and the reality of the adoption [3,13], but also probably due to shared major challenges with normative parenthood [27,28]. Likewise, previous studies found that both groups of parents may present similar levels of parenting stress [22], which, as suggested by our findings, may be related to the high and similar levels of parental difficulties shared by the groups in the aforementioned domains.

Surprisingly, adoption-related difficulties were one of the three less frequent types of parental difficulties reported by adoptive parents. Several authors assume that, globally, adoptive parents are aware of the potential occurrence of adoption-specific challenges but have developed adequate coping strategies to deal with these issues, preventing them to reach critical levels, perhaps as a result of the formal preparation to adopt [44,45]. However, in our sample, as expected, parental difficulties related to parent–child relationship and children’s developmental/emotional/behavioral problems were high and more frequent among adoptive parents than among biological ones. As such, we could argue that adoptive parents may not be fully aware of the influence that a child’s previous adversities may have on the child’s behavior and on the quality of parent–child relationship, rather overvalue the influence of the experiences lived in the adoptive family [34] and minimizing the perceptions of such kind of difficulties. Likewise, the fact that some adoptive families considered themselves as more similar than different to conventional families [34,35] may support this explanation.

Finally, although previous studies argue that the need for post-adoption supportive services appears to increase over time [13,45], the most intense parental difficulties faced by adoptive parents in our sample emerged during the first year after adoptive placement, a trend that was not observed among biological parents. This is consistent with research suggesting that the first-year post-placement can be particularly demanding for adoptive parents [2], as they often need to deal with unmet children’s expectations [44]; besides, this finding contradicts prior evidence suggesting that the parental experiences of adopting a child may be comparable to those of biological parents after birth [29].

### 4.2. Help-Seeking

Our findings added to prior research showing that adoptive parents sought professional help more frequently than biological ones [23,30], even after controlling for parental difficulties. This finding suggests that help-seeking may be mainly related to the family type (biological vs. adoptive) rather than to the greater difficulties experienced by adoptive parents. This is in line with previous literature: adoptive parents had usually characteristics considered to be advantageous to seek professional help compared to biological parents (i.e., they are typically older, with higher education and financial stability) [2]; in addition, most of they have developed multiple strategies to handle their previous experiences of infertility and all have undergone an in-depth evaluation process for adoption [21], during which they were continuously alerted for the importance of their ability to manage stress, access to support systems and invest in self-care [46]. We could expect that these factors may have made adoptive parents more aware of the benefits of help-seeking than biological parents.

Adoptive parents may also easier legitimate the need to ask for professional help due to their children’s prior adversities, without questioning their own parental resources, as previous studies suggest that adoptive parents are likely to have made positive evaluations of themselves as a parent [23]. However, according to our findings, it is more reasonable to expect that some adoptive parents may be more concerned about their own parenting abilities than biological ones [10], namely when confronted with unexpected children’s characteristics [44] and having difficulties in recognizing whether their family’s difficulties reflect the child’s developmental stage or adoptive status [47]. This may also explain their greater demand for professional help, even in the absence of severe family problems. Interestingly, in our sample, although difficulties related to parents’ well-being and normative parental challenges were the most frequent among adoptive and biological parents, they were only associated with help-seeking among adoptive parents. Adoptive parents often had less support from other adoptive parents with whom they can share their parenting challenges and feel understood [9]. They may also not feel comfortable to share their parental concerns with biological parents of their social network, which prevent them from recognizing universal sources of parental stress as well as the potential impact of parenting on parents’ well-being.

### 4.3. Barriers and Satisfaction

The most prevalent barriers to seeking professional help faced by adoptive parents were in line with previous research on this topic: perceived lack of problems and reduced services’ awareness are common barriers related to post-adoption help-seeking [11,14,35,37]. However, despite a trend towards a greater prevalence of those barriers among adoptive than biological parents, these differences did not reach statistical significance. A similar non-significant pattern was observed for attitudinal barriers. The generalization of these results deserves particular attention, as they may be influenced by the asymmetry between groups (with fewer adoptive parents reporting not seeking help compared to biological ones), which, coupled with the multiple variables controlled for in the models, may have reduced power for the detection of significant effects. Similar constraints need to be considered regarding parents’ satisfaction with the professional help received. Adoptive and biological parents reported similar levels of satisfaction, but future research is needed to replicate these findings with more homogeneous samples.

However, an interesting and possibly related result was that the more the adoptive parents reported parental difficulties related to children’s problems, the less they were satisfied with the support received. We could argue that adoptive parents are likely to access generalist rather adoption-specialized services to deal with their children’ problems in our country, which are not always perceived by parents as competent in adoption-related issues [15]. This explanation is likely to be supported by our findings, as adoption-specialized help-seekers in our sample faced greater difficulties related to their own well-being and adoption-specific issues, but similar difficulties related to their children’s behavioral difficulties, when compared to adoptive parents who sought non-specialized professional support. In line with this, despite only 41% of adoptive parents have sought adoption-specialized help, these parents perceived the professional support received as more useful than those who requested non-specialized help. This result is in accordance with research showing that adoptive parents greatly value adoption-competent professionals and services [15,26,38]. However, our results also demonstrated that the more adoptive parents who sought for adoption-specialized help reported difficulties related to the quality of the parent–child relationship, the less satisfied they were with the help received. This suggests that current adoption specialized services may be not suited to those parents’ specific difficulties in the country.

### 4.4. Acceptability of Psychological Parenting Interventions

Our results demonstrated, on average, a high and similar perceived utility of future psychological parenting interventions aimed to promote parents’ own well-being and the quality of the parent–child relationship among adoptive and biological parents, whereas the former reported a greater availability to participate in such interventions. An interesting finding is that the group effect on parents’ availability to participate become non-significant after controlling for group’s parental difficulties. Therefore, while help-seeking seems to vary based on family type more than based on parental difficulties, the availability to participate in future psychological parenting interventions seems to be dependent on parental difficulties, a finding that mirrors prior conclusions concerning the influence of parenting-related challenges on parents’ intention to participate in future parenting interventions [43]; this also indicates that remediative interventions may have a greater acceptability than preventive ones.

Additionally, as the experience of specific types of difficulties (e.g., related to their own well-being, the parent–child relationship, normative parenting challenges) were related to higher perceived utility among adoptive parents, but not among biological ones, it may be also plausible to expect that psychological parenting interventions targeting the aforementioned areas may be of greater value for adoptive parents. Regardless of having sought specialized or non-specialized professional support, adoptive parents continue to report a high availability to participate in future psychological parenting interventions. This may be due to the more psychosocial nature of the support provided by adoption services in the country and, on the other hand, to the absence of psychological parenting interventions with this scope that may be sensitive to adoptive parents’ needs.

### 4.5. Limitations and Future Research

Several limitations of this study should be acknowledged, namely: (1) its cross-sectional design, (2) data collection through an online survey and possible associated selection bias (i.e., parental difficulties concerns/awareness were likely to be higher among participating parents, only those with access to Internet could participate), (3) the asymmetry and reduced size of the subgroups of adoptive and biological parents (who did and did not seek professional support) and of adoptive and biological male participants, and (4) the used self-report measures specifically developed for this study, due to the absence of others widely used in previous research that could enable us to achieve our specific goals.

Overall, further research is warranted to replicate and generalize these exploratory findings and then, inform conclusive policy and practice recommendations. Longitudinal studies are recommendable to provide a more comprehensive picture of how parental difficulties and help-seeking may interact over time. The consideration of other variables related to adoptive families (e.g., parents’ attributions of their children’ behaviors, specific family developmental transitions) and help-seeking (e.g., types of services, waiting times, costs, availability of informal sources of support) would allow testing some of the explanations for the results found in this study (e.g., higher propensity to professional help-seeking among adoptive vs. biological parents). Besides, parents’ acceptability of future psychological parenting interventions should be assessed more deeply in future studies, by specifying the intervention background, format and features, in order to directly inform the design of programs according to parents’ needs and preferences. After structured governmental post-adoption services are available in Portugal, further research will be also needed to analyze which barriers prevent parents to seeking adoption-specialized professional help and whether the needs of support are met by them.

### 4.6. Implications for Practice

Despite its exploratory nature, our study suggests important implications for practice. First, echoing prior advices [38], they reinforce the need for post-adoption specialized services in the country, which should be focused on promoting issues related, not only with the child and the parent–child relationship but, in particular, to help parents to manage their own well-being and normative parenting challenges, which is in line with prior recommendations [3,4,13,18]. Importantly, this study stresses the priority of such support be available soon after adoptive placement. The provision of high-quality preadoption services [44,45], coupled with specialized multifaceted services during the first stages of the post-adoption period, may promote families’ successful adjustment before serious difficulties reach a critical point. It is important to note that even difficulties similar to those presented by biological parents are expected to have different etiologies [3] and, thus, to require different approaches among adoptive parents in order to be effective.

Besides, as psychological parenting interventions were reportedly acceptable to adoptive parents, this is an area of development to be considered by policy makers, as previously suggested by other authors [4,16,18]. Particularly, the findings of this study suggest that adoptive parents may benefit from psychological parenting interventions—rather prevention strategies, as the availability to participate was dependent on higher levels of parental difficulties—targeting, in particular, their own well-being, the parent–child relationship, and normative parenting challenges, which can be offered complementary to other post-adoption services [6].

Finally, in order to overcome the identified barriers to seeking professional help, our results reinforce the urge for agencies to adopt a proactive rather than a reactive approach to post-adoption support [10,11], at two levels. On one hand, in line with prior recommendations [10,14,37] and taking advantage of the high propensity of adoptive parents to seeking professional support, agencies need to inform parents about which services are available and how to access them. Importantly, it is worth to explore the extent to which this communication/awareness may be improved by considering community partnerships beyond the child welfare system (e.g., school, health care) [24]. On the other hand, professionals need to be prepared to help parents to differentiate normative behaviors from those possibly requiring professional intervention. Along with improving professionals’ and parents’ education about adoption issues [39], special training and awareness on problems’ severity recognition could be particularly helpful.

## 5. Conclusions

Our study added to prior research on post-adoption by privileging a comparative and multidimensional approach to parental difficulties and its associated help-seeking behaviors and perceptions, thus expanding the scarcity and inconsistency of past research on this topic. Importantly, it comprises one of the largest and up-to-date sample of adoptive parents in Portugal, thus enabling to draw solid implications for the design of post-adoption support in the country, while inspiring research and practice in other countries. Specifically, the knowledge gained in Study 1 contributes to inform post-adoption services development regarding adoptive parents shared and specific needs, behaviors and perceptions regarding help-seeking, when compared to biological ones. Our findings also contribute to clarify how the professional help currently available in the Portuguese community is perceived by adoptive parents and their acceptability of future specific interventions. The knowledge gained in Study 2 contributes to identify the needs and perceptions currently associated with adoption-specialized help-seeking, clarifying in which extent they differ from those presented by adoptive parents who asked for non-specialized help.

## Figures and Tables

**Table 1:** Sample Characteristics of Study 1, According to Family Type (Adoptive vs. Biological Parents): Descriptive Statistics and Group Comparisons.

Study Variables	Total Sample *N* = 1023 *n* (%)	Adoptive Parents ^a^ *N* = 471 *n* (%)	Biological Parents ^a^ *N* = 552 *n* (%)	*t*/χ^2^
Parents-related				
Age (years); *Mean* (*SD*; *Range*)	42.25 (7.12; 21–66)	46.97 (5.18; 34–66)	38.22 (5.99; 21–54)	−25.04 ***
Sex				
Male	242 (23.7)	193 (41.0)	49 (8.9)	145 ***
Female	781 (76.3)	278 (59.0)	503 (91.1)	
Education				
Elementary/High school	288 (28.2)	153 (32.5)	135 (24.5)	8.10 **
University/Postgraduate degree	735 (71.8)	318 (67.5)	417 (75.5)	
Professional status				
Employed	930 (90.9)	442 (93.8)	488 (88.4)	9.09 **
Unemployed or other	93 (9.1)	29 (6.2)	64 (11.6)	
Marital status				
Single	138 (13.5)	54 (11.5)	84 (15.2)	3.36
Widower	6 (0.6)	3 (0.6)	3 (0.5)	
Separated/Divorced	74 (7.2)	37 (7.9)	37 (6.7)	
Married/Cohabitating	805 (78.7)	377 (80.0)	428 (77.5)	
Number of household members; *Mean* (*SD*; *Range*)	3.45 (0.88; 1–8)	3.39 (0.93; 1–8)	3.49 (0.82; 1–6)	1.92
Number of biological and/or adoptive children; *Mean* (*SD*; *Range*)	1.56 (0.74; 0–1)	1.54 (0.78; 1–6)	1.57 (0.69; 0–4)	0.73
Mental health problems				
Never had	750 (73.3)	372 (79)	378 (68.5)	14.51 **
Had in the past	183 (17.9)	68 (14.4)	115 (20.8)	
Currently has	90 (8.8)	31 (6.6)	59 (10.7)	
Physical health problems				
Never had	811 (43.5)	366 (77.7)	445 (80.6)	3.15
Had in the past	72 (7.0)	31 (6.6)	41 (7.4)	
Currently has	140 (13.7)	74 (15.7)	66 (12)	
Infected with COVID-19				
Never	988 (96.6)	463 (98.3)	525 (95.1)	7.85 ***
Yes, in the past	31 (3.0)	7 (1.5)	24 (4.3)	
Yes, currently	4 (0.4)	1 (0.2)	3 (0.5)	
Risk population for COVID-19				
No	890 (87.0)	418 (88.7)	472 (85.5)	2.36
Yes	133 (13.0)	53 (11.3)	80 (14.5)	
Perceived impact of COVID-19; *Mean* (*SD*; *Range*) ^b^	2.60 (0.42; 1–5)	2.65 (0.39)	2.55 (0.44)	−3.96 ***
Children-related				
Age; *Mean* (*SD*; *Range*)	7.90 (4.70; 0–17)	9.87 (3.71; 1–17)	6.22 (4.81; 0–17)	−13.68 ***
Sex				
Male	531 (51.9)	247 (52.4)	284 (51.4)	0.10
Female	492 (48.1)	224 (47.6)	268 (48.6)	
Adoption-related				
Application type				
Single		68 (14.4)		
Couple		403 (85.6)		
Children in the adoptive family				
Only adopted children		387 (82.2)		
Both adopted and biological children		84 (17.8)		
Number of children adopted at the same time; *Mean* (*SD*; *Range*)		1.19 (0.48; 0–4)		
Child’s age at adoptive placement (years); *Mean* (*SD*; *Range*)		4.12 (2.91; 0–15)		
Time since child’s adoptive placement (years); *Mean* (*SD*; *Range*)		5.83 (3.38; 0–17)		

*Note*. ** *p* < 0.01; *** *p* < 0.001. ^a^ Reference category for family type: 0 = Biological Parents. ^b^ The perceived impact of COVID-19 was measured through a bipolar adjective scale in which higher and lower values indicated, respectively, a greater positive and a greater negative perceived impact of COVID-19 in the participants’ lives.

**Table 2:** Characteristics of Adoptive Parents According to the Type of Help-seeking (Adoption-Specialized vs. Non-Specialized): Descriptive Statistics and Group Comparisons.

Study Variables	Total Sample *N* = 200 *n* (%)	Adoption Specialized Help-Seekers ^a^ (*n* = 82) *n* (%)	Non-Specialized Help-Seekers ^a^ (*n* = 118) *n* (%)	*t*/χ^2^
Parents-related				
Age (years); *Mean* (*SD*; *Range*)	46.93 (4.87; 35–59)	47.11 (4.88; 37–58)	46.81 (4.87; 35–59)	−0.44
Sex				
Male	74 (37.0)	43 (52.4)	67 (56.8)	0.37
Female	126 (63.0)	39 (47.6)	51 (43.2)	
Education				
Elementary/High school	48 (24.0)	23 (28)	25 (21.2)	1.25
University/Postgraduate degree	152 (76.0)	59 (72.0)	93 (78.8)	
Professional status				
Employed	191 (95.5)	76 (92.7)	115 (97.5)	2.57
Other	9 (4.5)	6 (7.3)	3 (2.5)	
Marital status				
Single	26 (13.0)	10 (12.2)	16 (13.6)	0.79
Widower	1 (0.5)	0	1 (0.8)	
Separated/Divorced	17 (8.5)	7 (8.5)	10 (8.5)	
Married/Cohabitating	156 (78.0)	65 (79.3)	91 (77.1)	
Number of household members; *Mean* (*SD*; *Range*)	3.54 (1.04; 2–8)	3.50 (1.02; 2–8)	3.56 (1.06; 2–7)	0.40
Number of biological and/or adoptive children; *Mean* (*SD*; *Range*)	1.65 (0.81; 1–6)	1.62 (0.83; 1–6)	1.67 (0.81; 1–5)	0.41
Mental health problems				
Never had	141 (70.5)	57 (69.5)	84 (71.2)	3.01
Had in the past	38 (19.0)	13 (15.9)	25 (21.2)	
Currently has	21 (10.5)	12 (57.1)	9 (42.9)	
Physical health problems				
Never had	147 (73.5)	55 (67.1)	92 (78.0)	4.59
Had in the past	18 (9.0)	7 (8.5)	11 (9.3)	
Currently has	35 (17.5)	20 (24.4)	15 (12.7)	
Infected with COVID-19				
Never	196 (98.0)	80 (97.6)	116 (98.3)	0.14
Yes, in the past	4 (2.0)	2 (2.4)	2 (1.7)	
Yes, currently	0	0	0	
Risk population for COVID-19				
No	176 (88.0)	74 (90.2)	102 (86.4)	0.66
Yes	24 (12.0)	8 (9.8)	16 (13.6)	
Perceived impact of COVID-19; *Mean* (*SD*; *Range*) ^b^	2.63 (0.38; 1.50–3.86)	2.58 (0.41; 1.50–3.86)	2.66 (0.34; 1.63–3.30)	1.50
Children-related				
Age; *Mean* (*SD*; *Range*)	10.81 (3.04; 2–17)	11.34 (2.88; 5–17)	10.43 (3.10; 2–17)	−2.10 *
Sex				
Male	110 (55.0)	34 (41.5)	40 (33.9)	1.19
Female	90 (45.0)	48 (58.5)	78 (66.1)	
Adoption-related				
Application type				
Single	35 (17.5)	14 (17.1)	21 (17.8)	0.02
Couple	165 (82.5)	68 (82.9)	97 (82.2)	
Children in the adoptive family				
Only adopted children	158 (79.0)	70 (85.4)	88 (74.6)	3.40
Both adopted and biological children	42 (21.0)	12 (14.6)	30 (25.4)	
Number of children adopted at the same time; *Mean* (*SD*; *Range*)	1.25 (0.49; 0–3)	1.32 (0.49; 1–3)	1.20 (0.48; 0–3)	−1.62
Child’s age at adoptive placement; *Mean* (*SD*; *Range*)	4.76 (2.98; 0–15)	5.69 (2.79; 1–15)	4.11 (2.95; 0–15)	−3.78 ***
Time since child’s adoptive placement; *Mean* (*SD*; *Range*)	6.06 (3.06; 0–14)	5.64 (2.83; 1–13)	6.35 (3.19; 0–14)	1.61

*Note*. * *p* < 0.05; *** *p* < 0.001. ^a^ Reference category for type of support asked for: 0 = Non-specialized help-seekers. ^b^ The perceived impact of COVID-19 was measured through a bipolar adjective scale in which higher and lower values indicated, respectively, a greater positive and a greater negative perceived impact of COVID-19 in the participants’ lives.

**Table 3:** Specific Types of Parental Difficulties by Family Type (Adoptive vs. Biological Parents): Descriptive Statistics and Group effects.

Specific Types of Parental Difficulties	Adoptive Parents *N =* 471 M (*SE*)	Biological Parents *N* = 552 M (*SE*)	*F*
Parents’ well-being	2.65 (0.08)	2.67 (0.07)	0.03
Parent–child relationship	2.17 (0.08)	1.73 (0.07)	12.82 ***
Normative parenting challenges	2.39 (0.08)	2.17 (0.07)	3.20
Couple relationship	1.89 (0.09)	2.55 (0.08)	25.57 ***
Co-parenting	1.57 (0.09)	1.99 (0.09)	8.81 **
Children’s developmental/emotional/behavioral problems	1.99 (0.08)	1.27 (0.07)	34.82 ***
Children’s deviant behavior	1.35 (0.07)	0.38 (0.06)	96.31 ***
Children’s sexualized behavior	0.40 (0.04)	0.19 (0.04)	10.61 **
Children’s physical health problems	0.77 (0.06)	0.39 (0.06)	15.90 ***
Communication with external contexts to the family	1.18 (0.07)	0.94 (0.06)	5.99 *
Adoption-related difficulties	0.90 (1.19)		

*Note 1.* * *p* < 0.05; ** *p* < 0.01; *** *p* < 0.001. *Note 2*. The estimated marginal means and *F*’s values refer to the effect of family type on each specific type of parental difficulties after controlling for parents’ sex, age, education, professional status, mental health problems, perceived impact of COVID-19 pandemic and child’s age (Analysis of Covariance models).

**Table 4:** Barriers to Seeking Professional Help to Deal with Parental Difficulties, According to Family Type (Adoptive vs. Biological Parents): Descriptive Statistics and Group Effects.

Barriers to Seeking Professional Help	Adoptive Parents *n* = 51 *n* (%)		Biological Parents *n* = 198 *n* (%)		*B* (*SE*)
	No	Yes	No	Yes	
Not knowing who could help	36 (70.6)	15 (29.4)	146 (73.7)	52 (26.3)	0.78 (0.46)
Doubts about severity of the difficulties	25 (49.0)	26 (51.0)	89 (44.9)	109 (55.1)	−0.21 (0.41)
Not feeling comfortable about asking for help	45 (88.2)	6 (11.8)	139 (70.2)	59 (29.8)	−1.29 (0.56) *
Being ashamed to admit these difficulties	48 (94.1)	3 (5.9)	161 (81.3)	37 (18.7)	−1.27 (0.74)
Beliefs that no one will be able to help	43 (84.3)	8 (15.7)	154 (77.8)	44 (22.2)	−0.47 (0.54)
Fear of the “bad parent” label	39 (76.5)	12 (23.5)	149 (75.3)	49 (24.7)	0.14 (0.48)
Not wanting family/children to be negatively labeled	41 (80.4)	10 (19.6)	153 (77.3)	45 (22.7)	0.16 (0.51)
The partner did not want to ask for help	47 (92.2)	4 (7.8)	166 (83.8)	32 (16.2)	−1.11 (0.67)
Normalization of their difficulties	11 (21.6)	40 (78.4)	45 (22.7)	153 (77.3)	0.15 (0.49)
Financial difficulties to attend consultations	44 (86.3)	7 (13.7)	123 (62.1)	75 (37.9)	−1.14 (0.56) *
Lack of time for consultations	42 (82.4)	9 (17.6)	118 (59.6)	80 (40.4)	−1.25 (0.49) *
Fear of being penalized for missing work	49 (96.1)	2 (3.9)	150 (75.8)	48 (24.2)	−2.14 (0.86) *
No one to take care of the children during the consultations	48 (94.1)	3 (5.9)	142 (71.7)	56 (28.3)	−1.23 (0.70)
The child did not know about the adoption	49 (96.1)	2 (3.9)			

*Note 1.* * *p* < 0.05. *Note 2.* The *B*’s values refer to the effect of family type on each barrier to seeking professional support after controlling for parents’ sex, age, education, professional status, mental health problems, perceived impact of COVID-19 pandemic and child’s age (Multivariate Logistic Regression models). The distribution of the frequencies does not contemplate any correction considering the effect of the co-variates in the model, since it is not available for this type of analyses.

**Table 5:** Adoptive Parents’ Difficulties by Type of Help-seeking (Adoption-Specialized vs. Non-Specialized Help): Descriptive Statistics and Group Effects.

Specific Types of Parental Difficulties	Adoption Specialized Help-Seeking *n* = 82 *M* (*SE*)	Non-Specialized Help-Seeking *n* = 118 *M* (*SE*)	*F*
Parents’ well-being	3.40 (0.13)	3.05 (0.11)	4.18 *
Parent–child relationship	3.03 (0.16)	2.71 (0.13)	2.39
Normative parenting challenges	3.12 (0.15)	2.99 (0.12)	0.45
Couple relationship	2.44 (0.17)	2.19 (0.15)	1.23
Co-parenting	2.08 (0.19)	1.78 (0.16)	1.48
Children’s developmental/emotional/behavioral problems	3.16 (0.15)	3.17 (0.12)	0.01
Children’s deviant behavior	2.58 (0.18)	2.17 (0.15)	2.95
Children’s sexualized behavior	0.79 (0.14)	0.71 (0.12)	0.22
Children’s physical health problems	1.18 (0.16)	1.02 (0.14)	0.51
Communication with external contexts to the family	1.97 (0.16)	1.79 (0.13)	0.79
Adoption-related difficulties	1.52 (0.14)	1.09 (0.12)	5.16 *

*Note 1.* * *p* < 0.05. *Note 2.* The estimated marginal means and *F*’s values refer to the effect of type of help-seeking on each type of parental difficulties after controlling for parents’ sex, age, education, professional status, mental health problems, perceived impact of COVID-19 pandemic and child’s age (Logistic regression models).

## Data Availability

The data presented in this study are available on request from the corresponding author. The data are not publicly available due to ethical reasons.
